# 

**Published:** 1897-02-13

**Authors:** 


**Notices of Births, Deaths, and Marriages** are inserted in this column at a charge of 2s. 6d. for an announcement not exceeding four lines.

### NOTICE TO CORRESPONDENTS.

All MS., Letters, Books for Review, and other matters intended for the Editor should be addressed THE EDITOR, "THE HOSPITAL," THE HOSPITAL BUILDING, 28 & 29, SOUTHAMPTON STREET, STRAND, W.C.

The Editor cannot undertake to return rejected MS., even when accompanied by stamped directed envelope.

**Notice to Advertisers and Subscribers.**

All Advertisements, Orders for Copies of THE HOSPITAL, and Business Communications should be addressed to **THE MANAGER, (Not to The Editor),** THE HOSPITAL, The Hospital Building, 28 & 29, Southampton Street, Strand, London, W.C.

The Cost of Subscription, post free, is as follows:—Per week, 2½d.; per quarter, 2s. 8d.; per half-year, 5s. 3d.; per annum, 10s. 6d. Foreign:—Per annum, 15s. 2d.; payable, in all cases, in advance.

**NOTICE.—Vol. XX. of "The Hospital" (April to September, 1896), in handsome cloth binding, is now on sale at the Office of this Journal, price 7s. 6d. Cases for binding the Numbers contained in this Volume, 1s. 6d. Index to Vol. XX., 2½d., post free.**

**The Monthly Part for February is now ready, Price 6d., by post 8d.**

"THE HOSPITAL" LIST OF ANNUAL SUBSCRIBERS.

*Banker's Order Form.*

The Prince of Wales's Hospital Fund for London.

Joint Stock Bank, Cheque Bank, Trustees' Savings Bank, Post Office Savings Bank, or a deposit account at one of the Stores.

(Fill in the Name of your Bank.)

To............................................................

PLEASE PAY to the Bank of England, to the credit of the above named Fund, the sum of £ s. d. on the First day of February, 1897, and on or after the First day of February in every future year till further notice.

Signature............................................................
Full Name, &c., for postal purposes ............................................................
Address ............................................................
............................................................
............................................................

£

Our readers who desire to respond to this appeal will fill up and cut out this form and send it to the Manager, The Scientific Press, 28 and 29, Southampton Street, Strand, London, W.C. Envelopes should be marked on the outside "Prince of Wales's Hospital Fund."

### The Student of Medicine.

The following questions were set at the examination held in January for the triple qualification granted by the Royal Colleges of Edinburgh and the Faculty of Physicians and Surgeons of Glasgow:—

Anatomy (Five Years' Course). All the questions to be answered.

1. Describe the capsular ligament of the shoulder-joint: name the muscles which are in direct contact with it, and give the nerve-supply of each.
2. Describe the superficial fascia over the lower part of the anterior wall of the abdomen, and over the anterior half of the perineum, and state their dispositions in the scrotum.
3. Describe the femoral artery in the middle third of the thigh.
4. Describe the third cranial nerve in the orbit.

Advanced Anatomy (Third Examination—Five Years' Course). All the questions to be answered.

1. What bones come into relation to the superior longitudinal and lateral sinuses of the skull when traced in the direction of the course of the blood?
2. Describe the course and relations of the median nerve from the bend of the elbow to the anterior annular ligament.
3. Mention in order from above downwards the structures in relation to the deep surface of the gluteus maximus.
4. Describe the left ventricle of the heart, its position on the surface of the heart, the relative position of its openings, and the structure and arrangement of its auriculo-ventricular valve.

Physiology (All the questions to be answered).

1. Describe and contrast the functions of (*a*) the cerebral lobes; (*b*) the cerebellum; (*c*) the spinal cord.
2. From what substances are urea, uric acid, and hippuric acid derived, and where are they formed?
3. Give an account of the microscopical, chemical, and physical properties of blood.
4. What is "peristaltic action"? By what mechanism is it produced, and how is it controlled?

Pathology (All the questions to be answered).

1. To what morbid changes is the muscular tissue of the heart-wall liable? Describe the appearances of the lesions you mention.
2. Distinguish, as regards cause and structure, between interstitial and vesicular emphysema of the lungs.
3. Describe the histology of a cylinder or columnar celled epithelioma, and say where such tumours are most commonly found.
4. Give a short account of the characters and life-history of the typhoid bacillus (Eberth's). In what parts of the human body may this organism be found?

